# Identifying Causative Agents of a Paretic Syndrome in Waterbirds in Southern Portugal

**DOI:** 10.3390/toxins17020062

**Published:** 2025-01-31

**Authors:** María V. Mena Casero, Andrew D. Turner, Begoña Ben-Gigirey, Ryan P. Alexander, Karl J. Dean, Robert G. Hatfield, Benjamin H. Maskrey, Christelle Mazuet, Kobey Karamendin, Rafael Mateo

**Affiliations:** 1 Wildlife Rehabilitation and Research Center of Ria Formosa (RIAS), Rua do Parque Natural da Ria Formosa, 8700-194 Olhão, Portugal; 2 Instituto de Investigación en Recursos Cinegéticos (IREC), Junta de Comunidades de Castilla-La Mancha (JCCM), Consejo Superior de Investigaciones Científicas CSIC)—Universidad de Castilla-La Mancha (UCLM), Ronda de Toledo 12, 13005 Ciudad Real, Spain; rafael.mateo@uclm.es; 3 Centre for Environment Fisheries and Aquaculture Science (Cefas), The Nothe, Barrack Road, Weymouth DT4 8UB, UK; andrew.turner@cefas.gov.uk (A.D.T.);; 4 Centro Nacional Instituto Español de Oceanografía (IEO-CSIC), Centro Oceanográfico de Vigo, 36390 Vigo, Spain; 5 Centre National de Référence des Bactéries Anaérobies et Botulisme, Institut Pasteur, Université Paris Cité, F-75015 Paris, France; christelle.mazuet@pasteur.fr; 6 Scientific and Production Center of Microbiology and Virology, 105 Bogenbay Batyr Street, Almaty 050010, Kazakhstan; kobey.karamendin@gmail.com; 7 Institute for Environmental Assessment and Water Research (IDAEA-CSIC), Jordi Girona 18, 08034 Barcelona, Spain

**Keywords:** paretic syndrome, botulism neurotoxin, toxins, etiology, diagnosis

## Abstract

Paretic and paralyzing syndromes affecting wild birds are widely described in the literature, with outbreaks showing an increase in frequency and intensity worldwide during recent years. In the Iberian Peninsula, a paretic clinical picture without known etiology affecting mostly gulls has been reported during the last few decades. This paretic syndrome (PS) affects waterbirds and is characterized by a set of signs of ascendent flaccid paralysis, dyspnea, and diarrhea at different levels of severity. This study presents the first macro-analysis of some potential etiological PS agents in wild birds in southern Portugal. Other possible etiologies of PS related to nutritional deficiencies and environmental pollutants were not studied but are also discussed here. A total of 571 samples, belonging to 377 individuals with (*n* = 336) and without (*n* = 41) PS signs, have been tested for seven different toxins groups (botulinum neurotoxin (BoNT), paralytic shellfish toxins (PSTs), domoic acid (DA), anatoxin-a (ATX-a), cylindrospermopsin (CYN), tetrodotoxins (TTXs), and microcystins (MCs)) and three viral infections (gull adenovirus (GA), Newcastle disease virus (NVD), and highly pathogenic avian influenza viruses (HPAIV)). Of all the birds tested for botulinum neurotoxin, those with PS signs were positive (100%) and those without PS signs were negative (0%), confirming an association between PS and botulism. Some samples were positive for PSTs and MCs, but the prevalence in birds with PS signs was not significantly higher (2.5% and 5.3%, respectively) than in birds without signs (5.4% and 5.4%, respectively). Two birds without PS signs were positive for highly pathogenic avian influenza virus. The presence of the rest of the toxins and viruses was negative for all the samples tested. Our results support the relevant contribution of botulinum neurotoxin in the PS outbreaks observed in several species of aquatic birds in the last decades in southern Portugal, suggesting it could be one of the main causes of mortality in waterbirds.

## 1. Introduction

**Figure 1:**
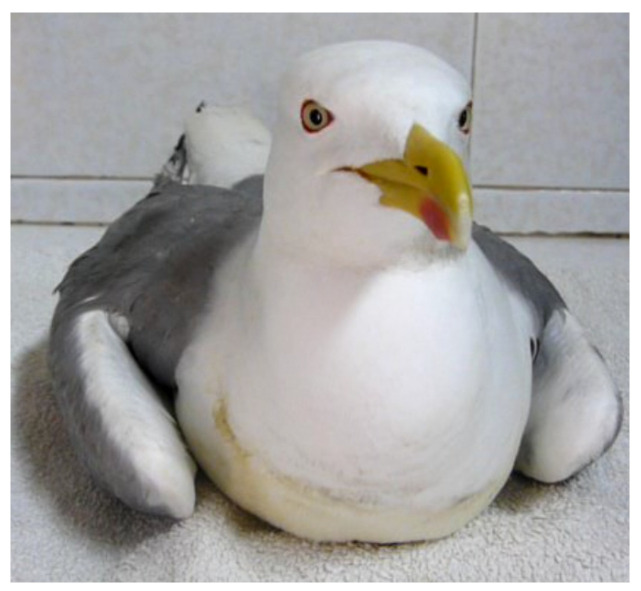
Gull with paretic syndrome.

Paralytic outbreaks in wild birds have shown an increase in frequency and intensity during the last few decades, being described as a cause of mass mortality and disease events worldwide and becoming one of the main threats to waterbirds (Figure 1) [1,2,3,4,5,6,7].

There are several etiologies of biotic (e.g., toxins, viruses) and chemical (e.g., anthropogenic pollutants) origin that can cause paralytic conditions in wild birds [8,9,10,11]. Sonne et al. [2] divided the etiologies of bird paralysis into four groups: infectious diseases (bacteria, viruses, fungi), toxins (e.g., paralytic shellfish toxins, botulinum neurotoxins), environmental contaminants (neurotoxic chemicals), and nutritional (vitamin and mineral deficiencies). The identification of the etiology of paralytic outbreaks in waterbirds must be based on the observation of clinical signs and analytical tests (i.e., presence of pathogens, toxins, environmental pollutants, or specific biomarkers), but the diagnosis can be complex because signs are not unique to a single etiology, and the presence of a causative agent does not always imply its contribution to the illness. The following paragraphs summarize the etiologies more frequently involved in paralytic conditions in birds.

Different avian viruses have been identified as causing disease and mortality in wild populations [12,13,14,15,16,17], and some of them present signs compatible with PS, like diarrhea, depression, anorexia, respiratory distress, and neurological alterations. The most relevant are paramyxoviruses (i.e., Newcastle disease), herpesviruses (i.e., Marek’s disease), influenza viruses (i.e., avian influenza), and adenoviruses. Newcastle virus disease (NVD) causes respiratory and neurological signs like dyspnea, paralysis, and opisthotonos [16]. Marek’s disease is characterized by a progressive leg and wing paralysis, opisthotonos, weight loss, dyspnea, eye lesions, diarrhea, anorexia, and death [17]. Highly pathogenic avian influenza (HPAI) signs can range from subclinical to death, including depression, anorexia, and respiratory and neurologic signs [13]. Adenovirus infections, such as the recently described gull adenovirus (GA), can be subclinical or cause respiratory and neurologic signs and high mortality events [15]. The detection of avian viruses can be performed by molecular techniques such as PCR, offering the most specific and sensitive results in a wide variety of samples [12,13].

Toxins have been identified as one of the main causes of disease and mortality in freshwater and marine wild birds worldwide [8,9,18,19,20,21,22,23,24,25]. Paralytic toxins like botulinum neurotoxin (BoNT), paralytic shellfish toxins (PSTs), domoic acid (DA), brevetoxins, tetrodotoxins (TTXs), and anatoxin-a (ATX) could be part of the suspected etiologies for PS in waterbirds. Moreover, other hepatotoxic cyanotoxins (cylindrospermopsin (CYN) and microcystins (MCs) can develop neurological signs because of the hepatic failure and, also, because of their cooccurrence with other cyanobacteria species producing neurotoxins [22]. Different methods can be used to identify and quantify toxins: biological, chemical, immunological, instrumental, or functional. Their use will depend on the type of toxin and the objective of the diagnostic. Analytical techniques such as high-performance liquid chromatography–tandem mass spectrometry (HPLC-MS/MS) allow high specificity in detection, as well as quantification at very low concentrations [25].

Among the environmental pollutants, heavy metals such as lead and mercury and anthropogenic contaminants like organochlorines and organophosphates also cause signs compatible with PS, among them diarrhea, paresis, ataxia, convulsions, weakness, and death [2]. Analytical chemistry is well developed for the detection and quantification of inorganic and organic environmental pollutants, and experimental and field studies with birds offer the information necessary for the interpretation of the detected levels [26].

Finally, the deficiency of vitamins A, B, and E and calcium has also been described as a cause of clinical signs similar to PS in wild birds. Vitamin B deficiency causes anorexia, ataxia, paralysis, and convulsions [1,27,28,29]. Deficiency of vitamin D causes paralytic signs [27]. The lack of vitamin C causes weakness, cramping, spasms, paresthesia, and carpopedal seizures [27]. Moreover, synergies between vitamin B deficiency and botulism can occur due to the thiaminase activity of some *Clostridium botulinum* strains [2,30]. Deficiency of vitamins and minerals can be suspected by the symptoms and confirmed by measuring blood or tissue levels. The response to treatment can also help to confirm a suspect [27].

In the Iberian Peninsula, a paretic syndrome (PS) has been reported in waterbirds during the last decades. This paretic affection is characterized by different severity levels of ascendent flaccid paralysis, dyspnea, and diarrhea that affect wild birds linked to aquatic ecosystems, mainly gulls but also ducks, coots, and waders. This work aimed to conduct the first macro-analysis of possible etiologies of PS in wild birds in southern Portugal, which included the study of the occurrence of five groups of marine and freshwater neurotoxins (BoNT, PSTs, DA, ATX-a, and TTXs), two groups of hepatotoxins (CYN and MCs), and three viruses (HPAI, NVD, and GA). The epidemiological associations between the presence of PS outbreaks and some potential etiological agents were analyzed to identify those causing PS in the studied waterbirds. Other etiologies of PS, like nutritional deficiencies and environmental pollutants, are also discussed.

## 2. Results

Laboratory results confirmed the presence of BoNT, PSTs, MCs, and HPAI and the absence of DA, ATX-a, CYN, TTXs, GA, and NDV in the samples tested. The presence of the BoNT-encoding gene was detected in all the pools from birds with PS signs tested for this neurotoxin (100%) and was absent in all the pools of birds without PS signs (0%, *p* < 0.001), assuming that all the birds in a pool from the same outbreak died from the same etiology (Table 1). For the total number of birds (with and without syndrome), the presence of the BoNT-encoding genes was higher in waders, ducks, and coots (100%) than in gulls (31.3%). In fact, BoNT-encoding gene prevalence was 100% in all the groups of birds with PS syndrome, including gulls (Table 1). The BoNT-encoding gene PCR revealed the presence of mosaic type C/D BoNT with toxicity confirmed by a mouse bioassay (MBA) in all positive cases. No quantification was performed in BoNT analysis. One pooled sample of maggots obtained from bird carcasses was also positive for the BoNT-encoding gene.

The presence of PSTs was confirmed in 6 individuals (3.1% of the 195 tested) (Table 1). The group with PS symptoms showed 4 positives (2.5%), and the group without PS signs showed 2 positives (5.4%) (Table 1). No statistically significant differences in the prevalence between both groups were found (*p* = 0.319). The PSTs concentrations found ranged between 5.5 and 8.7 µg STX di-HCl eq/kg. The presence of MCs was confirmed in 10 (5.3%) of the 188 individuals tested (Table 1). The birds with PS symptoms showed 8 positives (5.3%), and the group without symptoms revealed 2 positives (5.4%) (Table 1). No statistically significant differences in prevalence were found between both groups of samples (*p* = 0.979). The concentrations of total summed MCs ranged from 1.6–30.2 µg/kg. HPAI was confirmed in 2 samples of birds without PS symptoms, so no association was observed with the PS. None of the individuals, neither those with PS signs nor those without PS signs, tested positive for DA, ATX-a, CYN, TTX, GA, or NVD. All these results by four groups of birds (gulls, waders, ducks, coots, and others) are summarized in Table 1.

Regarding the type of sample, those that tested positive for PSTs were livers, and for MCs were livers, one kidney, and one cloaca content sample. Only one sample tested positive for more than one toxin (BoNT and MCs) belonging to an *L. michahellis* liver with PS signs.

## 3. Discussion

Our results confirmed BoNT type C/D as the most likely cause of PS in waterbirds in southern Portugal. These results are in accordance with those found by other authors, evidencing botulism as one of the main causes of death in aquatic birds in the Iberian Peninsula and elsewhere [9,30,31,32,33,34,35,36]. Reports of botulism in wild birds in southern Portugal have not been confirmed until now, but the presence of *C. botulinum* in wetlands and outbreaks of botulism in wild birds have been widely studied in southern Spanish wetlands during the last decades [31,32,33,34,35,36,37,38]. Although all bird species are theoretically susceptible to botulism, it is known that different sensitivities may exist among taxonomical groups of birds [34,39]. While necrophagous species, flamingos and divers appear to be resistant to the toxin, waterfowl and shorebirds seem to be the most susceptible groups [34,39]. Waterfowl, coots, waders, and gulls are among the most affected groups [33,34,35,39,40,41,42,43,44,45,46]. Here, we found the BoNT-encoding gene in all the tested individuals of these groups of birds with PS.

Other biotic contaminants, like microalgae and cyanobacteria, are known producers of several neurotoxins that can cause a clinical picture compatible with PS in birds [10,23,24,25,47,48,49,50,51,52]. However, our results showed no statistical relationship between the presence of PSTs and MC and PS signs. The seasonal variability of DA and PSTs occurrence in mollusks in southern Portugal found by Lima et al. [53] is also similar to the PS outbreaks in birds recorded at the Wildlife Rehabilitation and Research Centre—RIAS (Centro de Recuperação e Investigação de Animais Selvagens), but this can be simply because the factors that contribute to the appearance of marine toxins like DA and PSTs are similar to those contributing to the development of BoNT, for example, the temperature [37].

The information in literature about toxic levels of marine toxins in wild birds is limited. A wide range of PST levels has been described in wild bird samples, ranging from 1.4 µg/kg bw to 850 µg/kg bw [11]. Piatt et al. [54] set values between 1.4 and 3.9 µg/kg bw in common guillemot samples as trace levels, possibly without clinical consequences. By contrast, Starr et al. [55] considered PST levels between 10 and 85 µg/kg bw as compatible with the death in wild birds, and Mons et al. [56] established the oral LD50 of PSTs in pigeons at 91–100 µg/kg bw. Taking into consideration these findings, PST levels in the present study between 5.5 and 8.7 µg/kg bw can be considered sublethal. Regarding MC, Takahashi and Kaya [57] determined the LD50 of 256 µg/kg bw in quails, and Chen et al. [58] analyzed liver samples of waterbirds during a MC surface bloom, finding concentrations between 18 and 30 ng/g dry weight. Therefore, the levels detected in the present study, ranging from 1.6 to 30.2 µg/kg, can be considered sublethal.

One liver sample was positive for the presence of both BoNT and MCs (7.4 µg/kg bw). Murphy et al. [59] also detected an outbreak involving MCs, ATX, and BoNT in the same samples, underlying the possibility of multiple toxins involved in outbreaks. In these cases, the identification of the primary etiology of mortality may be more difficult to perform without a quantitative assessment of the toxin presence and a pathologic examination of the birds. Therefore, synergistic effects between toxins or even with other etiologies must be considered [59,60,61,62].

Other potential etiologies of PS, like nutritional deficiencies and environmental pollutants, must also be discussed. Thiamine deficiency has been suggested as a potential cause of PS in gulls [1], but this may not explain all the PS outbreaks observed in waterbirds [28]. Some strains of *C. botulinum* and some blue-green algae can have thiaminase activity [2,28,63,64]. In *C. botulinum* type A, thiaminase I structure and activity have been characterized [64]. Furthermore, several authors treated botulism-affected humans, mice, and birds with vitamin B1 with clinical success, indicating the positive response of botulism cases to thiamine supplementation [1,65,66]. The birds analyzed in this study were treated with a support therapy that included nutritional complement with vitamin B complex (Duphalyte^®^, with 0.10 mg/mL of thiamine) at a dose of 25 mL per bird once at admission. Having into consideration that the treatment for thiamine deficiency causing neurological signs includes the administration of 1–50 mg/kg SID at least for one week [66,67,68,69,70,71,72], the amount administered at admission (2.5 mg of thiamine) would not be enough to explain the successful recovery observed in the patients treated.

In relation to heavy metals and anthropogenic contaminants causing signs compatible with PS, mercury has the capacity to bioaccumulate in seabirds and can cause neurologic disorders, but the concentrations commonly detected in gulls of the south Atlantic coast of the Iberian Peninsula are below the levels associated with neurotoxicity [73].

## 4. Conclusions

Disease and mortality events affecting coastal birds are difficult to study and have been historically underdiagnosed and/or considered caused by marine toxins [8,74]. Focusing on PS events, diagnosis related to toxins is difficult to achieve due to the complexity, specificity, and high costs of the analyses required. Furthermore, gull species have been frequently considered as pests and have been subjected to population control measures [75,76,77], which reduced the research interest in mortality events affecting these groups of birds. However, because of the increase and expansion of biotoxin outbreaks in recent decades, in part associated with climate change [37,78,79,80,81,82] and their potential relationship with the decline of gull populations in Europe and elsewhere [5,83], the occurrence of botulism in gulls has begun to receive more attention over the last decades [33,43,44,46,84]. Surveillance and monitoring of wildlife diseases is a priority in conservation biology and One Health [74,79,85], particularly understanding the influence of global warming and anthropogenic activities in the mortalities caused by toxins [53,81].

## 5. Materials and Methods

### 5.1. Study Area and Waterbird Groups

All the samples and data analyzed were collected at RIAS, located in Olhão, Southern Portugal. A total of 7862 birds linked to aquatic ecosystems with PS were admitted at RIAS between January 2010 and December 2023 (Table 2), being this one of the main causes of admission at the hospital. Birds received alive (*n* = 5528) were diagnosed with PS, based on the presentation of different levels of ascendent flaccid paralysis, dyspnea, and diarrhea. Birds admitted dead (*n* = 2334) had the same diagnosis based on the necropsy findings and the date of arrival concurring with a PS outbreak. Bird samples (*n* = 571; lung, kidney, liver, intestine, and cloaca content/feces) were obtained during necropsies of some of the birds with PS (*n* = 336) and with other conditions (control group, *n* = 41) admitted either dead or dying after admission. Information about the species analyzed is given in Table S1. Moreover, a sample of fly larvae removed from a set of decomposing carcasses was also collected. In summary, 227 individuals were tested for one or more toxins, and 179 individuals were tested for one or more viruses.

### 5.2. Botulinum Neurotoxin Analysis

**Figure 2:**
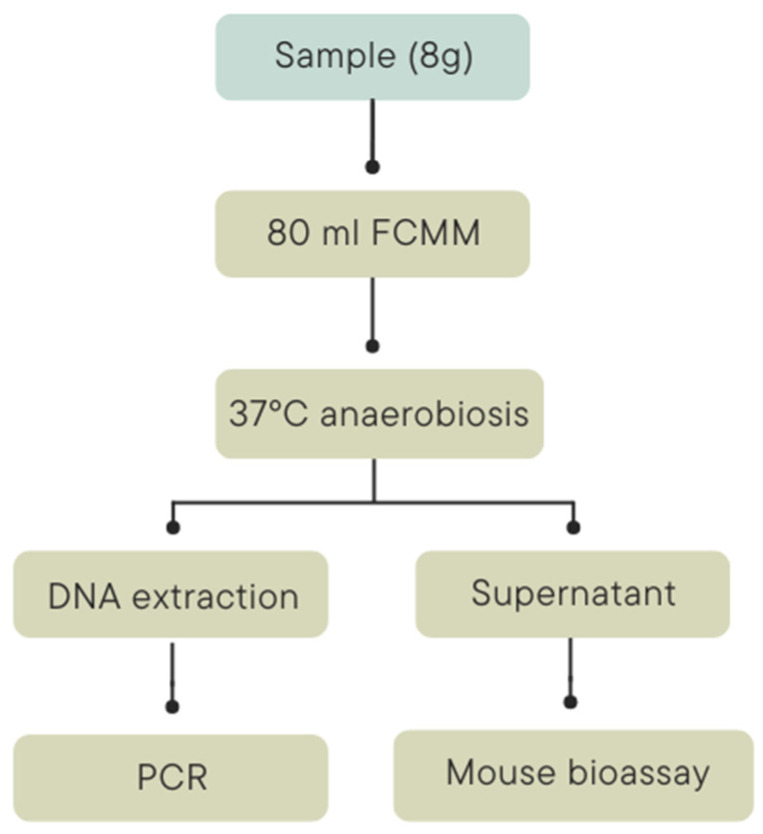
Analyses performed for *Clostridium botulinum* detection and botulinum neurotoxin detectionat the National Reference Center for anaerobic bacteria and botulism (Centre National de Référence des Bactéries Anaérobies et du Botulisme) in the Pasteur Institute (Paris, France).

Bird samples (liver and intestine; *n* = 66) belonging to 33 individuals and one pool of maggots were analyzed for BoNT at the National Reference Centre for Anaerobic Bacteria and Botulism (Centre National de Référence des Bactéries Anaérobies et du Botulisme) in the Pasteur Institute (Paris, France) (Table 3). The bird samples were placed in pools of 5–6 individuals, livers and intestines separately (Table 1) [86]. The analysis undertaken for the detection of BoNT or its encoding gene included mouse bioassay (MBA) and real-time PCR (RT-PCR) targeting the gene encoding BoNT C/D and E, respectively. In both cases, samples were analyzed after an enrichment culture (Figure 2).

Pooled samples of liver and intestinal contents (8 g) were diluted in fortified-cooked meat medium (FCMM; BD Difco, Jersey, NJ, USA) and incubated at 37 °C ± 2 °C in anaerobic conditions (Anoxomat Mark II; 90% N_2_/5%H_2_/5% CO_2_) [87,88]. After 48 h of incubation, 1 mL of enrichment culture was collected, and DNA was extracted using the QIAamp DNA Stool Kit (Qiagen, Courtaboeuf, France) according to the manufacturer’s instructions [87,88,89]. Detection of *C. botulinum* toxinotype C, C/D, D, D/C, and/or E in the extracted DNA was performed by SYBR green RT-PCR targeted to neurotoxin genes with primers P1652 and P1653 for the bont/C gene, P1795 and P1796 the bont/CD gene, P1654 and P1655 for the bont/D gene, P1797 and P1798 for the bont/DC gene, and P1650 and P1651 for the bont/E gene [85,86,87]. RT-PCR was performed in a total volume of 25 µL containing 12.5 µL of 2× concentration of I Taq Universal SYBR Green SuperMix (Bio-Rad, Los Angeles, CA, USA), 5 pmole of each primer, 5 µL of template DNA, and 7.3 µL of ultrapure water (B. Braun, Melsungen, Germany). Amplifications were performed on a CFX96 Real-Time System (Bio-Rad) using 96-well microwell plates. A PCR positive for bont/C means the presence of a type C strain; positive for bont/C and bont/CD means a type C/D, positive for bont/D means a type D, positive for bont/D and bont/DC means a type D/C, and positive for bont/E means a type E.

Detection of BoNT in the 96 h enrichment culture of the pooled samples was performed with the mouse lethality bioassay. The tests were performed according to European Directive 2010/63/EU on the protection of animals used for scientific purposes (laboratory animal use agreement no. 2013-0116). Enrichment broth (1 mL) was collected, centrifuged, filtered, and diluted (1:5) in 50 mM phosphate buffer (pH 6.5) containing 1% gelatin. A volume of 0.5 mL was injected intraperitoneally into Swiss mice weighing 20–22 g (Charles River Laboratories, l’Arbresle, France). The mice were observed for up to 4 days for the presence of typical clinical signs (pinching of the waist, labored breathing, and paresis) and euthanized immediately after observation of such signs.

### 5.3. Algal and Cyanobacterial Toxin Analysis

A total of 262 bird samples (12 cloaca contents, 123 kidneys, and 127 livers) of 158 individuals with PS signs were tested for the presence of PSTs, DA, TTX, ATX-a, CYN, and MCs at the Centre for Environment, Fisheries and Aquaculture Science (CEFAS, Weymouth, UK), Weymouth, UK. In addition, 37 bird samples (4 cloaca contents, 11 kidneys, 11 intestines, and 11 livers) of 11 individuals were tested for the presence of PSTs and DA at the Vigo Oceanographic Centre of the Centro Nacional Instituto Español de Oceanografía (IEO-CSIC) (Vigo, Spain). Furthermore, 63 bird samples (kidneys and livers) of 37 individuals without PS signs were also analyzed at CEFAS as a control group (Table 3).

All the chemicals used were LC-MS-reagent grade or HPLC-reagent grade. Certified reference materials (CRMs) for purified toxin standards of DA, saxitoxin (STX), gonyautoxins 1–5 (GTX1–5), neosaxitoxin (NEO), decarbamoylsaxitoxin (dcSTX), N-sulfocarbamoyl gonyautoxin-2 and 3 (C1 and 2), decarbamoylneosaxitoxin (dcNEO), and decarbamoylgonyautoxin-2 and 3 (dcGTX2 and 3) were obtained from the Institute of Biotoxin Metrology, National Research Council Canada (NRCC, Halifax, NS, Canada), and from CIFGA Laboratorio S.A. (Lugo, Spain). Additional non-certified reference material standards of N-sulfocarbamoyl gonyautoxin-1 and 4 (C3 and 4), decarbamoylgonyautoxin-1 and 4 (dcGTX1 and 4), and gonyautoxin-6 (GTX6) were obtained from NRCC. A reference standard for deoxydecarbamoylsaxitoxin (doSTX) was obtained from Cawthron Natural Compounds (CNC; Nelson, New Zealand). Tetrodotoxin (TTX) CRM was obtained from CIFGA Laboratorio S.A. Cyanotoxin standards, including the microcystin (MC) analogues MC-RR, MC-LA, MC-LY, MC-LF, MC-LW, MC-YR, MC-WR, MC-HilR, MC-HtyR, MC-LR, and Asp3-MC-LR, together with Nodularin (NOD), ATX-a, and CYN, were all obtained from Enzo Life Sciences, Exeter, UK. A certified standard of [Dha7]-MC-LR was obtained from the Institute of Biotoxin Metrology, NRCC. Reference standards were used to prepare working calibration standards for external calibration purposes following standardized procedures [90,91,92,93].

At CEFAS, PSTs and TTXs analyses were performed by ultra-high-performance liquid chromatography—hydrophilic interaction chromatography—tandem mass spectrometry (UHPLC-HILIC-MS/MS) based on the method described by Boundy et al. [94] and validated by Turner et al. [91,95]. In brief, tissue samples were extracted with 1% acetic acid to give a solvent/sample ratio of 10:1 [96]. Samples were boiled, cooled, centrifuged, and desalted as described by Boundy et al. [94], prior to dilution and analysis. A Waters Acquity UPLC I-Class Waters coupled to a Xevo TQ-S tandem quadrupole mass spectrometer, and an Agilent Infinity II UHPLC coupled to an Agilent 6495B MS/MS were used for analysis. Chromatography was conducted using a 1.7 µm, 2.1 mm × 150 mm Waters Acquity BEH Amide UPLC column with a Waters VanGuard BEH Amide guard cartridge. LC-MS/MS instrument parameters were as described by Turner et al. [91,95]. Multiple Reaction Monitoring (MRM) transitions used for PST/TTX acquisition were those reported previously and as summarized in Table S2 [91,92]. Quantitation was conducted against the response factors calculated for all PSTs present in the six-point calibration standards available as certified reference standards. Toxicity equivalence factors (TEFs) for STX, NEO, dcSTX, dcNEO, dcGTX2 and 3, GTX1-6, C2, and C4 were taken from EFSA recommendations [97]. TEFs for other congeners C1, C3, dcGTX1 and 4, and doSTX were taken from other published evidence [91]. Individual toxin concentrations and total sample toxicity were calculated as described by Turner et al. [95].

At IEO-CSIC, PST analyses were carried out by HPLC with post-column oxidation and fluorescence detection (HPLC-PCOX-FLD) following Rourke et al. [98], with some modifications [99]. Homogenization and extraction protocols were adapted to seabird tissue samples [11]. Ten kidney samples (pre-cut with small scissors) and one cloacae content sample were individually homogenized using a small IKA Ultra-Trurrax^®^ Blender. Whenever possible, 5 g of sample was taken for analysis. Sample extraction and deproteination were carried out according to Van de Riet et al. [90]. In the case that sample weight was less than 5 g, extraction solvent volume was scaled down. After deproteination, supernatants were filtered through 0.22 µm PTFE syringe filters into vials ready to be analyzed by HPLC-PCOX-FLD. An Acquity UPLC system (Waters Corporation, Cerdanyola del Vallès, Spain) equipped with a binary solvent manager, column heater, sample manager, and an FLD detector was employed. Data acquisition and processing were performed using the Empower 3 software (Waters Corporation, Milford, CT, USA). The PCOX reaction system consisted of two Reagent Manager pumps (Waters Corporation) that delivered post-column reagents, a reaction coil (Teflon tubing 0.25 mm i.d., 8 m long), and a water bath capable of maintaining the coil temperature at 65 °C. Post-column reagents, column, mobile phases, gradient conditions, and FLD detector wavelengths employed were as described in Rodríguez et al. [99]. In order to determine the PSTs concentration in the samples, the external standard calibration procedure was used. The potential presence of naturally fluorescent compounds was checked as in Ben-Gigirey et al. [100].

Extraction of tissues for cyanotoxin analysis (MCs and Nod) performed at CEFAS followed the method of Turner et al. [92]. In brief, samples were extracted with 80% aqueous methanol to give a solvent-to-sample ratio of 10:1. Sample/solvent mixtures were thoroughly homogenized, centrifuged, and filtered into glass LC-MS autosampler vials before analysis in a Waters Acquity UPLC I-Class coupled to a Waters Xevo TQ-S tandem quadrupole mass spectrometer (Waters Corporation, Manchester, UK). Chromatography was conducted using a 1.7 µm, 2.1 × 50 mm Waters Acquity UHPLC BEH C18 column (P/N 186002350, Lot no. 0249343351) in conjunction with a Waters VanGuard BEH C18 1.7 µm 2.1 mm × 5 mm guard cartridge (P/N 186003975, Lot no. 0245343321). Instrumental conditions were as described by Turner et al. [92]. MRM transitions were built into the MS/MS method using positive mode acquisition for each toxin. Parent and daughter ions, all in positive ion mode, as well as cone and collision voltages, are summarized in Table S2. Quantification was performed against calibration curves prepared with the certified reference standards, and results were given in µg/kg of tissue.

The desalted, carbon-SPE cleaned-up acetic acid extracts of tissue samples were also analyzed at CEFAS for ATX, CYN, and DA using an in-house unpublished method. An Agilent 1290 Infinity II UHPLC module was used for chromatographic separation with the same HILIC column type and guard cartridge utilized for PST/TTX analysis. Detection was conducted using an Agilent 6495B triple quadrupole (MS/MS) with Jet Stream technology and electrospray with positive ionization. Source conditions were those used for PST/TTX analysis. MRM transitions and associated collision energies are summarized in Table S2.

DA was also determined in bird samples at IEO-CSIC facilities. Samples were minced with scissors and homogenized with an IKA Ultra-Trurrax^®^ blender. Then, 2 g of sample homogenate was weighed into 15 mL polypropylene centrifuge tubes, combined with 8 mL of 50% methanol, homogenized with the same blender for 2 min at medium speed, and centrifuged at 5400 rcf and 6 °C for 15 min. Supernatants were decanted and filtered through a 0.22 µm PTFE syringe filter. LC-high-resolution mass spectrometry analyses of the methanolic extracts were performed in a Dionex high-speed liquid chromatograph equipped with an Orbitrap mass analyzer and an ESI probe for electrospray ionization (Thermo Fisher Scientific, Waltham, MA, USA). The software used for MS analysis was Xcalibur 4.1 (Thermo Fisher Scientific). Analyses were conducted following De la Iglesia et al. [101], with a modified protocol. The column used for separations was a Kinetex 2.6 µm EVO C18 100 Å, LC Column 50 × 2.1 mm (Phenomenex Inc., Torrance, CA, USA). The mobile phase consisted of water/acetonitrile/formic acid (90.9:9.0:0.1) at a flow rate of 0.35 mL/min. Column and sampler temperatures were 40 °C and 5 °C, respectively. Run time was 6 min, and injection volume was 2 µL. The mass spectrometer was operated in positive ESI polarity. Source conditions were as follows: spray voltage +3700 V, capillary temperature 320 °C, sheath gas 40 arbitrary units (au), and aux gas 0 au. The instrument was set in full MS mode with the following parameters: scan range 250 to 400 *m*/*z*, mass resolution setting of 70,000, automatic gain control target of 106, maximum injection time of 200 ms. The extracted ion chromatograms within the 312–313 *m*/*z* range in positive mode were selected. A five-level calibration curve of DA dissolved in 50% methanol was prepared within the range of 0.3–10 µg/mL. The external standard calibration procedure was employed for quantification.

### 5.4. Virus Analysis

The presence of GA and NDV was studied at the laboratory of the Scientific and Production Centre of Microbiology and Virology (CMV) in Almaty, Kazakhstan. The presence of these two viruses was studied in the intestine of 23 birds with PS and 2 control birds (Table 3). For the search of GA presence, DNA was extracted using the RNA/DNA Extraction Kit (Invitrogen, Carlsbad, CA, USA, Thermo Fisher Scientific). PCRs were conducted using primers targeting the conserved hexon gene of the Aviadenovirus genus: 5′-GAYRGYHGGRTNBTGGAYATGGG-3′ (from FAdV-1 hexon gene nt positions 283–305, sense) and HeXR1: 5′-TACTTATCNACRGCYTGRTTCCA-3′ (from FAdV-1 hexon gene nt positions 1073–1051, antisense). PCR was performed by 35 cycles of 94 °C for 30 s, 55 °C for 30 s, and 72 °C for 30 s. The predicted size of the PCR products was approximately 800 bp [102]. For the NDV presence search, viral RNA was extracted from the field samples using the QIAamp Viral RNA Mini Kit (Qiagen) according to the manufacturer’s protocol. RT-PCR assays were performed using the OneTaq One-Step RT-PCR Kit (New England BioLabs [NEB]) according to the manufacturer’s instructions. Primers targeting the fragment of the fusion (F) gene were used [103].

HPAI presence was studied in cloacal and oropharyngeal swabs of 159 individuals between 2011 and 2023 at the national reference laboratory Instituto Nacional de Investigação Agrária e Veterinária (Lisbon, Portugal). Of those, 157 individuals showed PS signs (Table 3). These samples were collected within the Avian Influenza Passive Surveillance Plan of the Portuguese veterinary authorities (Direção Geral de Alimentação e Veterinária—DGAV), and subtyping the avian influenza virus by real-time RT-PCR was performed for H5, H7, and N1 presence.

### 5.5. Data Analysis

Birds were considered positive to an etiological agent when one of its samples was determined positive in the analyses described before. Then, the occurrence of the different etiological agents (BoNT, PSTs, MCs, and HPAI) was compared between birds with and without PS with a Pearson’s chi-squared test with Yates’ continuity correction of Fisher’s exact probability test. Significance was established at *p* < 0.05. The number of samples and individuals analyzed are summarized in Table 3. Statistical analyses were performed with IBM Statistics SPSS V28.0.

## Figures and Tables

**Table 1:** Results of the individuals analyzed for toxins and virus for the identification of causative agents of the paretic syndrome in the studied birds.

Etiological Agents		Group of Birds	PS + Group		PS − Group		Total	
			*n* (*p* *)	+	*n*	+	*n*	+
**Toxins**	Botulinum neurotoxin	Gulls	5 (1)	5 (1)	11 (2)	0	16 (3)	5 (1)
		Waders	6 (1)	6 (1)	0	0	6 (1)	6 (1)
		Ducks and coots	11 (2)	11 (2)	0	0	11 (2)	11 (2)
		Total	22 (4)	22 (4)	11 (2)	0	33 (6)	22 (4)
	Paralytic shellfish toxins	Gulls	140	4	28	1	168	5
		Waders	6	0	0	0	6	0
		Ducks and coots	12	0	1	0	13	0
		Others	0	0	8	1	8	1
		Total	158	4	37	2	195	6
	Domoic acid	Gulls	140	0	28	0	168	0
		Waders	6	0	0	0	6	0
		Ducks and coots	12	0	1	0	13	0
		Others	0	0	8	0	8	0
		Total	158	0	37	0	195	0
	Anatoxins	Gulls	133	0	28	0	161	0
		Waders	6	0	0	0	6	0
		Ducks and coots	12	0	1	0	13	0
		Others	0	0	8	0	8	0
		Total	151	0	37	0	188	0
	Cylindrospermopsin	Gulls	133	0	28	0	161	0
		Waders	6	0	0	0	6	0
		Ducks and coots	12	0	1	0	13	0
		Others	0	0	8	0	8	0
		Total	151	0	37	0	188	0
	Tetrodotoxins	Gulls	133	0	28	0	161	0
		Waders	6	0	0	0	6	0
		Ducks and coots	12	0	1	0	13	0
		Others	0	0	8	0	8	0
		Total	151	0	37	0	188	0
	Microcystins	Gulls	133	7	28	1	161	8
		Waders	6	1	0	0	6	1
		Ducks and coots	12	0	1	0	13	0
		Others	0	0	8	1	8	1
		Total	151	8	37	2	188	10
**Viruses**	Adenovirus	Gulls	17	0	0	0	17	0
		Waders	5	0	0	0	5	0
		Others	1	0	2	0	3	0
		Total	23	0	2	0	25	0
	HP avian influenza	Gulls	130	0	0	0	130	0
		Waders	2	0	0	0	2	0
		Ducks and coots	25	0	0	0	25	0
		Others	0	0	2	2	2	2
		Total	157	0	2	2	159	2
	Newcastle virus disease	Gulls	27	0	0	0	27	0
		Waders	6	0	0	0	6	0
		Others	1	0	2	0	3	0
		Total	34	0	2	0	36	0

* Number of pairs of pools (liver and intestine).

**Table 2:** Birds admitted at RIAS Wildlife Rehabilitation and Research Centre between 2010 and 2023 with paretic syndrome.

Group of Birds	Admitted Alive	Admitted Dead	Total
Gulls	4917	1047	5964
**Ducks and coots**	515	1274	1789
Waders	85	13	98
Others	11	0	11
**Total**	5528	2334	7862

**Table 3:** Samples analyzed for toxins and viruses in birds (*n* = 377) with (*n* = 336) and without (*n* = 41) paretic syndrome. Not all the samples or individuals were tested for all the etiological agents.

Etiological Agents/Laboratory		With PS		Without PS	
		Samples	Individuals	Samples	Individuals
**Toxins**	BoNT/Pasteur Institute	44	22	22	11
	PSTs/CEFAS and IEO-CSIC	266	158	63	37
	DA/CEFAS and IEO-CSIC	272	158	63	37
	ATX-a/CEFAS	251	151	63	37
	CYN/CEFAS	251	151	63	37
	TTX/CEFAS	251	151	63	37
	MCs/CEFAS	251	151	63	37
**Viruses**	HPAI/INIAV	159	159	2	2
	NVD/CMV	44	34	4	2
	NGA/CMV	23	23	4	2

## Data Availability

The original contributions presented in this study are included in the article/Supplementary Materials. Further inquiries can be directed to the corresponding author.

## Supplementary Materials

The following supporting information can be downloaded at: https://www.mdpi.com/article/10.3390/toxins17020062/s1, Table S1: Number of individuals analyzed for biotoxins and viruses of each species; Table S2: Mass spectrometer conditions used for the analysis of Paralytic Shellfish Toxins and cyanotoxins.
