# Supplementary material for: Identifying Causative Agents of a Paretic Syndrome in Waterbirds in Southern Portugal
Source: Toxins (Basel). 2025 Jan 31;17(2):62. doi: 10.3390/toxins17020062 (PMC11860645; doi:10.3390/toxins17020062)
Supplement: Supplementary file 1 [file toxins-17-00062-s001.zip › toxins-3358708-supplementary.pdf]

toxins

MDPI

Supplementary Materials: Identifying Causative Agents of a Paretic Syndrome in Waterbirds in Southern Portugal

María V. Mena Casero, Andrew D. Turner, Begoña Ben-Gigirey, Ryan P. Alexander, Karl J. Dean, Robert G. Hatfield, Benjamin H. Maskrey, Christelle Mazuet, Kobey Karamendin and Rafael Mateo

Table S1. Number of individuals analysed for biotoxins and viruses of each species.

Group of birds	Species	Individuals analysed
**Gulls**	*Larus fuscus*	162
	Larus michahellis	97
	Ichthyaetus audouinii	29
	Chroicocephalus ridibundus	18
	Stercorarius skua	1
	**Total**	**307**
**Anatids and Rallids**	*Anas acuta*	15
	Anas clypeata	15
	Anas crecca	5
	Anas platyrhynchos	2
	Anas strepera	2
	Aythya ferina	2
	Fulica atra	1
	Tadorna ferruginea	1
	Tadorna tadorna	1
	**Total**	**44**
Waders	*Arenaria interpres*	7
	Calidris alba	3
	Calidris alpina	2
	Charadrius alexandrinus	1
	Charadrius hiaticula	1
	Himantopus himantopus	1
	**Total**	**15**
**Others**	*Morus bassanus*	6
	Calonectris diomedea	2
	Phalacrocorax carbo	1
	Ardea cinerea	1
	Ciconia ciconia	1
	**Total**	**11**
	**Total**	**377**

Table S2. Mass spectrometer conditions used for the analysis of Paralytic Shellfish Toxins and cyanotoxins.

Analogue	ESI+ Transition	Cone (V), (CE (eV))	ESI- Transition	Cone (V), CE (eV)
STX	**300.1>204.1**,138.0	10 (23; 30)		
NEO	**316.1>126.1**,220.1	10 (26: 23)		
dcSTX	**257.1>126.1**,222.0	10 (19; 22)		
dcNEO	**273.1>126.1**,225.1	10 (20; 18)		
doSTX	**241.1>60.0**,206.1	10 (23; 22)		
TTX	**320.1>302.1**,162.1	40 (25; 38)		
GTX2			**394.1>351.1**, 333.1	10 (16, 24)
GTX3	396.1>298.1	10 (17)	**394.1>333.1**	10 (22)
GTX1			**410.1>367.1**,349.1	10 (15; 22)
GTX4	412.1>314.1	10 (18)	**410.1>367.1**	10 (15)
GTX5	**380.1> 300.1**	10 (16)	378.1>122	10 (25)
GTX6	**396.1> 316.1**	10 (15)	394.1>122	10 (25)
dcGTX2			**351.1>164.0**,333.1	10 (30; 17)
dcGTX3	**353.1>255.1**	10 (18)	351.1>333.1	10 (17)
dcGTX1			**367.1>274.1**,349.1	10 (20; 17)
dcGTX4	**369.1>271.1**	10 (18)	367.1>349.1	10 (17)
C1			**474.1>122.0**,351.1	10 (30; 25)
C2	**396.1>298.1**	18 (20)	474.1>122.0	10 (30)
C3	412.1>332.1	18 (16)	**490.1>410.1**	10 (20)
C4	**412.1>314.1**	18 (20)	490.1>,392.1	10 (20)
MC-RR	519.9 > **134.9**; 126.9; 102.8	30 (30; 50; 70)		
Nod	825.5 > **135.1**; 103.1	55 (60; 100)		
MC-LA	910.1 > **135.1**; 106.9	35 (70: 80)		
[Dha[7]]-MC-LR	981.5 > **135.0**; 106.8	75 (75: 80)		
[Asp3] MC-LR[a]	981.5 > **134.9**; 106.9	75 (70; 80)		
MC-LF	986.5 > **213.0**; 135.0	35 (60; 65)		
MC-LR	995.6 > **135.0**; 127.0	60 (70; 90)		
MC-LY	1002.5 > **135.0**; 106.9	40 (70; 90)		
MC-HilR*	1009.7 > **134.9**; 126.9; 106.9	75 (75; 90; 80)		
MC-LW	1025.5 > **134.9**; 126.8	35 (65; 90)		
MC-YR	1045.6 > **135.0**; 126.9	75 (75; 90)		
MC-HtyR	1059.6 > **134.9**; 106.9	75 (70; 90)		
MC-WR	1068.6 > **134.9**; 106.9	80 (75; 100)		
DA	312.1 > **266.1;** 161.2; 133.2	(14; 26; 34)		
ATX	166.1 > **149.1;** 131.1; 91.1	(14; 18; 30)		
CYN	416.1 > **336.2;** 194.1; 177.1	(26; 40; 40)		

Primary MRM for quantitation shown in bold; CE = Collision energy

Function The identifier for a group of MRM transitions acquired within a programmed window

Cone, V Cone voltage in volts

CE, eV Collision energy in electron volts

[a][Dha[7]]-MC-LR and [Asp3] MC-LR unresolved so reported together when detected
